# Impact of early follow-up CT in the conservative management of traumatic brain injury on surgical decision making: A retrospective, single-center analysis with special respect to coagulopathy

**DOI:** 10.1007/s00068-024-02449-3

**Published:** 2024-03-26

**Authors:** Mats L. Moskopp, Dag Moskopp, Lennart W. Sannwald

**Affiliations:** 1 TU Dresden Faculty of Medicine Carl Gustav Carus: Technische Universität Dresden Medizinische Fakultat Carl Gustav Carus, Dresden, Germany; 2 Department of Neurosurgery, Faculty of Medicine and University Hospital Carl Gustav Carus, Technische Universität Dresden, Fetscherstraße, Dresden, Germany; 3 Institute of Physiology, Medical Faculty Carl Gustav Carus, Technische Universität Dresden, Fetscherstrasse, Dresden, Germany; 4 Department of Neurosurgery, Vivantes Friedrichshain Hospital, Charité Academic Teaching Hospital, Landsberger Allee, Berlin, Germany; 5 Department of Health - Social Affairs - Education, European Technical College EUFH, Rolandufer, Berlin, Germany; 6 Department of Neurosurgery, University of Ulm, Bezirkskrankenhaus Günzburg, Lindenallee, Germany

**Keywords:** Traumatic brain injury, Early routine follow-up computerized tomography, Coagulopathy, Lesion size

## Abstract

**Introduction:**

Initial management of traumatic brain injury (TBI) without immediate need for surgical therapy varies across centers. The additional value of routine repeat cranial computerized tomography (CT) to neurological monitoring is controversial. This retrospective study investigates the impact of routine follow-up CT after 6 h (CT6h) in initially conservatively managed TBI on surgical decision making. Furthermore, the impact of coagulopathy on lesion size and progression was examined.

**Methods:**

We reviewed charts of patients admitted to our clinic in the time between 1st January 2020 and 30th June 2022 for the ICD10 diagnosis S06.3 (traumatic brain contusion), S06.4 (epidural hematoma), S06.5 (subdural hematoma), and S06.6 (traumatic subarachnoid hemorrhage). Baseline characteristics as well as timing, reason, and consequences of first and second cranial CT, clinical course, lesion size at first and second CT as well as presence and type of coagulopathy (standard laboratory testing and prior medical history) were noted among others. Significance testing was carried out using Student’s *t*-test. The significance level was set to *p* < 0.005.

**Results:**

A total of 213 patients were included, 78 were operated after first CT, 123 underwent clinical and imaging surveillance, and 12 patients were not treated. CT6h did not anticipate imminent neurological deterioration. Early secondary deteriorating patients (9/123, 7.3%) did so before 6 h after admission clustering between 3 and 4 h (6/9, 66.7%). CT6h changed surgical decision making in one case (1/114, < 1%). Nine out of 106 (8.5%) patients managed conservatively after CT6h showed a late secondary clinical deterioration or failure of conservative treatment, eight out of which had stable size of hemorrhage in CT6h. There was no significant difference in lesion size at first CT related to the presence of coagulopathy, antiplatelet agents, or anticoagulant drugs for SDH or contusions. In patients with radiological progression of SDH in combined brain injury (CBI), coagulopathy was associated with a higher increase of lesion size (diameter increase > 6 mm: 11.1% with vs. 2.8% without coagulopathy). This effect was not observed for contusions in CBI (volume increase > 6 ml: 17.4% with vs. 22.7% without coagulopathy).

**Conclusion:**

Early routine follow-up CT does neither anticipate imminent neurological deterioration nor impact surgical decision making. A substantial number of patients with initially stable follow-up imaging need delayed surgery due to conservative treatment failure. If patients can be monitored clinically, surgical decision making depends on clinical status. Patients with coagulopathy do not present with larger lesions, but show a higher ratio of drastic increase in SDH in contrast to contusions.

## Introduction

Care of patients with traumatic brain injury (TBI) has been one of the main pillars of modern neurosurgery and a major occupation for pioneers in neurosurgery such as Theodor Kocher and Harvey Cushing [1]. While an international standard of indications for surgical therapy was defined by the Brain Trauma Foundation (BTF) in 2006, the approach to early conservative management of TBI patients with intracranial pathology without the need for surgical therapy varies greatly from institution to institution [2–6].

Controversies regarding early conservative management predominantly evolve around the need for cranial computerized tomography (CT) screening with routine repeat CT versus repeat CT triggered by neurological deterioration only [7, 8]. While cranial CT is a quick, reliable, and widespread screening method for intracranial pathologies after trauma, the number of patients that deteriorate secondarily after surgical indications according to BTF guidelines were applied remains low.

Although approximately 20 to 35% of routine follow-up CTs after detection of intracranial bleeding show progressive brain injury, the therapeutic consequences are commonly linked to neurological status instead of imaging results [7, 9]. Change in treatment protocols after routine follow-up CT is regularly cited to occur in less than 1% of cases, which changes dramatically if linked to neurological worsening, leading to a change in treatment in up to 40% of cases [9–11]. However, the time interval to follow-up CT plays an important role as many studies report routine follow-up intervals of 12 h or more with up to an average of 38 h cited by Brown et al., thus increasing the likelihood that neurological deterioration occurs before repeat CT [9–12]. This creates a severe bias towards the impact on decision making of CTs triggered by neurological deterioration. In order to meaningfully change treatment protocols, routine follow-up CT must shortly precede and thus anticipate imminent neurological deterioration. If time interval to follow-up CT is too short, a significant change will only be observed in most extreme cases, while too long an interval is likely to miss patients that deteriorate neurologically before completion of the follow-up interval.

Due to potentially life-threatening consequences of intracranial pathologies, the threshold for repeat cranial CT remains low, possibly exposing many stable patients to unnecessary radiation and using a significant amount of CT resources in major trauma centers. While follow-up CT imaging has a high impact in moderate and severe TBI and in patients that cannot be monitored clinically, contribution to surgical decision making and clinical monitoring in patients with mild TBI and intracranial pathologies remains debatable.

In this retrospective, single-center study, we analyze the impact of an early follow-up CT after six hours (CT6h) algorithm for conservatively treated trauma patients with intracranial pathology on neurosurgical decision making in a level I trauma center in the capital of Germany.

## Methods

### Data collection

In this retrospective analysis, we included all patients with TBI and intracranial pathology who presented to our emergency department and were admitted to our neurosurgical department in a level I trauma center in central Berlin (Germany) between 1st January 2020 and 30th June 2022. For this, TBI must be the patient’s main diagnosis with a concomitant need for treatment or surveillance as indicated by intracranial pathology or neurological symptoms. Patients with incidental minor radiological findings of intracranial bleeds and concomitant major other traumatic sequelae are admitted to trauma surgery and not included in this study. Patient charts were filtered for ICD10 diagnosis S06.3 (traumatic brain contusion), S06.4 (epidural hematoma), S06.5 (acute and subacute subdural hematoma), and S06.6 (traumatic subarachnoid hemorrhage). No patients were excluded. For each patient, baseline parameters at admission such as age, sex, GCS, neurological deficit, coagulation disorder, trauma mechanism as well as results of cranial CT, surgical therapy or not, time interval between first and second CT, reason for second CT (routine or early due to neurological deterioration), consequence of second CT, whether more than two CTs were necessary in early management, the reason for further CTs (routine or clinical deterioration) and consequences of further CTs, were collected by chart review. The clinical courses of all patients that had delayed surgery were examined individually in more detail to evaluate the correlation of surgical decision making with the imaging algorithm described below. Images were reviewed by board-certified radiologists and confirmed by study authors. Coagulation disorder was defined as a positive history for intake of known antiplatelet drugs or anticoagulants as well as pathological laboratory testing according to standard values provided by the laboratory (< 150 platelets/nl, PT < 78%/INR > 1.2, aPTT > 38 s). The size of the injury was determined as a volume (width × height × length/2 for contusions) and largest diameter on axial CT imaging for extraaxial hematoma.

### Conservative TBI management algorithm

In our hospital, patients with head trauma are screened with cranial CT according to the Canadian CT head injury rule. Patients with abnormal initial CT and non-short-term GCS 3 to 8 due to seizures underwent either direct operation and/or ICP measurement. Due to the invasive nature of the ICP probe implantation, these cases were counted on the operative branch of this study. All patients with intracranial pathology without indication for surgery according to BTF guidelines are monitored in a critical care setting (GCS and neurological exam every hour at least) and receive a CT6h regardless of the underlying traumatic pathology. These patients were counted on the surveillance branch of this study. If imaging and neurological status remain stable, patients without the need for neurocritical care are transferred to regular wards on the next morning. In case of urgent need for ICU capacity, the earliest possible transfer of a stable patient to a normal ward is after CT6h. If patients deteriorate neurologically, an emergent CT will be performed. If patients remain neurologically stable but routine imaging shows a progressive brain injury, CT is repeated after 6–12 h. All patients with intracranial pathology receive 1 g tranexamic acid intravenously. Additionally, all patients with direct oral anticoagulants (DOAC) or vitamin K antagonists (VKA) receive 2.000 to 3.000 international units of prothrombin complex concentrate (PCC) for anticoagulant reversal depending on age and weight (25–35 units/kg). Lastly, all patients with a low prothrombin time/international normalized ratio receive 1 international unit PCC/kg per percent difference to 70% until the prothrombin time surpasses 70% (INR ≤ 1.2). Patients with single antiplatelet therapy are treated with tranexamic acid, while desmopressin (0.3 µg/kg intravenously) is reserved for increased intraoperative bleeding. Double antiplatelet therapy is met with tranexamic acid and desmopressin, whereas the need for platelet transfusions is discussed on a case-by-case basis with our team of hemostaseologists.

### Statistical analysis

The impact of CT6h was analyzed descriptively. The predictive value of CT6h for change in surgical decision making (number of surgical indications due to CT6h by amount of CT6h) as well as negative predictive value for later surgical therapy in case of CT6h without surgical consequence (number of late deteriorations by amount of CT6h without impact on surgical decision making) were calculated. Analysis of mean volume (contusions) or mean diameter (subdural hematoma) of intracranial pathology was carried out to detect differences according to the presence of coagulopathy. The difference of lesion size depending on the presence of coagulopathy was tested for statistical significance by a two-tailed Mann–Whitney U test. Significance testing was performed in GraphPad Prism 5 Software (version 5.01). Due to the relatively small sample size, the significance level was set to a *p*-value < 0.005 as proposed by Benjamin DJ et al. [13]. Patient consent was waived due to the pseudonymized retrospective nature of the study.

### Compliance with ethical standards

The results of this study were generated in the course of in-house quality control research about departmental care of patients with traumatic brain injury and as such were in accordance with the guidelines of the ethics committee of Ärztekammer Berlin.

## Results

### Patient distribution and characteristics

**Fig. 1:**
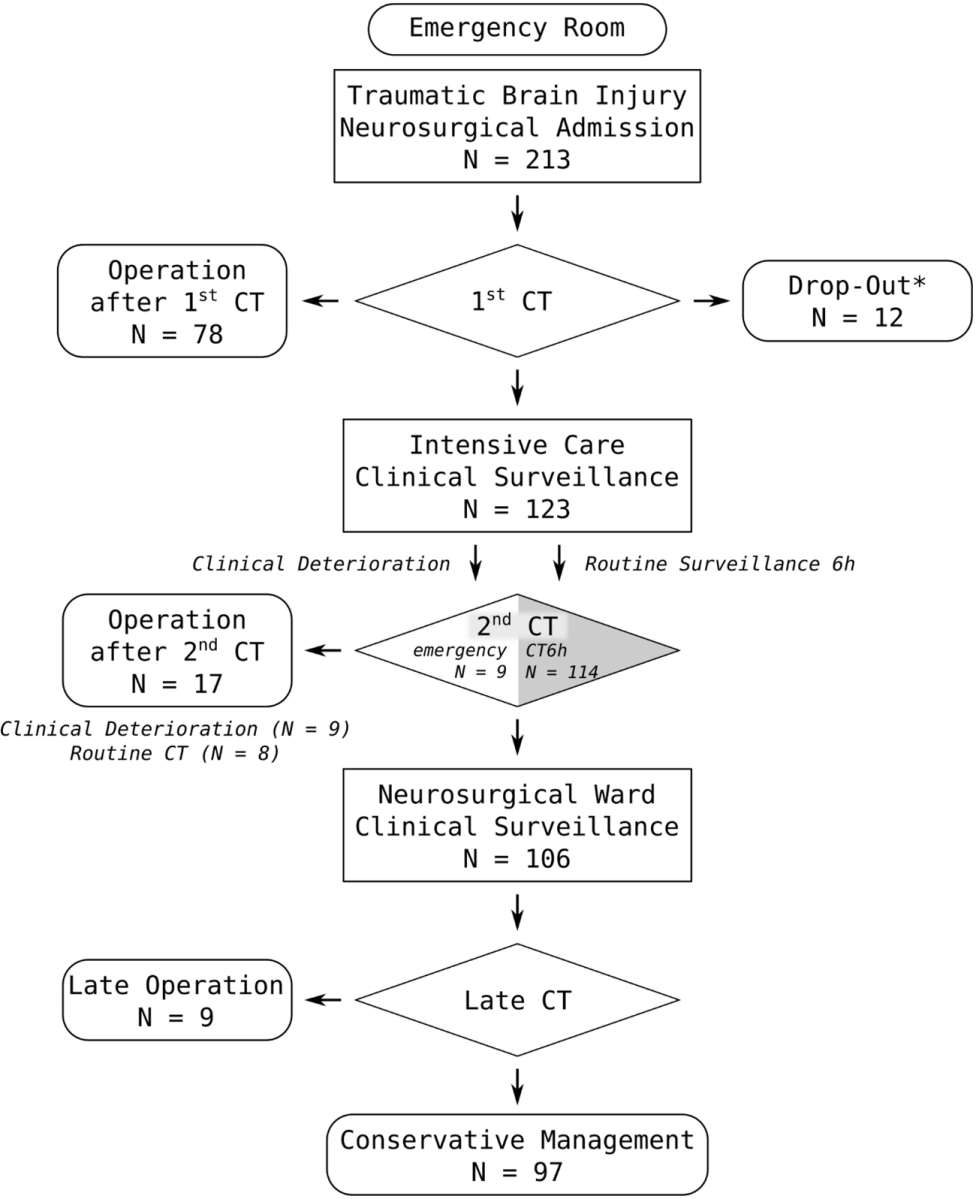
Treatment pathways. A total of 213 patients presented to the emergency room and were admitted to neurosurgery. After the first cranial CT, 78 were treated surgically, while 123 were monitored and had a CT6h. Therapy was limited in 12 patients (“Drop-Out*”). Nine of 123 monitored patients showed clinical deterioration, had emergency imaging before CT6h, and went on to surgery. The remaining 114 patients had CT6h, eight of which went on to have surgery that was planned after first CT already in seven cases (see text). A total of 106 patients received further conservative treatment after CT6h, nine of which showed later deterioration or failure of conservative treatment and went on to surgery after days to weeks. Ninety-seven of 213 patients received definitive conservative treatment

Overall, 213 patients with TBI and intracranial pathology in cranial CT were admitted to our neurosurgical department between 1st of January 2020 and 30th of June 2022. Their distribution across clinical pathways is shown in Fig. 1. Of these 213, 78 patients (36.6%) underwent immediate surgery. Another 12 patients (5.6%) did not receive any further treatment other than the best supportive care as was decided in consensus by the medical team and relatives. Thus, 123 (57.7%) patients were monitored clinically in an intensive care setting. The baseline characteristics of patients that underwent immediate surgery and patients that were monitored are displayed in Table 1.

**Table 1:** General characteristics of patient cohort. Left column: parameters, age in years, sex (male:female), Glasgow coma scale, neurological deficits. Middle column: patients that had surgery after the first CT (*n* = 78). Right column: the remaining patients that initially received conservative treatment

	Surgery after 1st CT	Surveillance + Drop Out
*N*	78	135
Age (years)	67.6 ± 18.7	64.3 ± 20.0
Sex (m:f)	1.8: 1	1.9: 1
Initial GCS*		
GCS 13–15	56/78 [71.8%]	114/135 [84.4%]
GCS 9–12	4/78 [5.1%]	12/135 [8.9%]
GCS 3–8	18/78 [23.1%]	8/135 [6.0%]^§^
Neurological deficit at presentation	45/78 [57.7%]	28/123 [22.8%]
No major deficit	16/78 [20.5%]	96/135 [71.1%]
Motor deficit^#^	35/78 [44.9%]	8/135 [5.9%]
Seizure^#^	1/78 [1.3%]	6/135 [4.4%]
Aphasia^#^	10/78 [12.8%]	2/135 [1.5%]
Behavioral changes^#^	15/78 [19.2%]	17/135 [12.6%]
Trauma mechanism		
Fall due to tripping	53/78 [67.9%]	87/135 [64.4%]
Fall from stairs	1/78 [1.3%]	15/135 [11.1%]
Fall from height (> 2 m)	1/78 [1.3%]	5/135 [3.7%]
Traffic accident	7/78 [9.0%]	17/135 [12.6%]
Violence	6/78 [7.7%]	7/135 [5.2%]

^*^ For the “Surveillance + Drop Out” group, in one case, no GCS was documented

^§^ Four were dropouts, three were postictal and improved rapidly, and one was intoxicated

^#^ Multiple items per patient were able to be detected as *neurological deficit at presentation*

In summary, mean age (67.6 ± 18.7 for immediate surgery and 64.0 ± 20.2 for initial conservative treatment) and sex distribution (male:female 1.8:1 for immediate surgery and 1.7:1 for initial conservative treatment) were similar. While the immediate surgery group showed a higher ratio of patients with GCS 3–8 (18/78 or 23.1% vs. 6/123 or 4.9%), most patients were GCS 13–15 in both groups (56/78 or 71.8% in immediate surgery vs. 105/123 or 85.4% in initial conservative treatment). Eight of 135 (5.9%) cases in the group *Surveillance* + *Drop Out* had a GCS of 3 to 8. These patients did not undergo invasive ICP monitoring for the following reasons: Of these eight, four were dropouts with no further treatment intended. Three were postictal and showed rapid clinical improvement. In one case, the patient showed a small SDH of only 3 mm, while alcohol blood levels indicated severe intoxication.

However, the immediate surgery group displayed neurological deficits more than twice as often (45/78 or 57.7% vs. 28/123 or 22.8%), motor deficits six times more often, and aphasia five times more often than initially conservative patients, whereas behavioral changes (12.2%) were the most prevalent changes in patients initially treated conservatively. In both groups, “fall due to tripping” was the most common trauma mechanism (67.9% in immediate surgery, 65.9% in initial surveillance), while fall from stairs (1.3% in surgery vs. 9.8% initial conservative treatment) and traffic accidents (9.0% in surgery vs. 13.0% initial conservative treatment) predominated in the conservative group. Also, the incidence of coagulation disorders was similar in both groups (53.8% in initial surgery vs. 50.4% in initial conservative treatment).

In patients that were immediately chosen for surgery, 11/78 (14.1%) had epidural hematoma, 65/78 (83.3%) suffered from subdural hematoma, 6/78 (7.7%) sustained traumatic brain contusions, and 14/78 (17.9%) had traumatic subarachnoid hemorrhage (multiple injuries per patient possible). In patients that were initially treated conservatively, 8/123 (6.5%) had epidural hematoma, 80/123 (65.0%) suffered from subdural hematoma, 52/123 (42.3) sustained traumatic brain contusions, and 68/123 (55.3%) had traumatic subarachnoid hemorrhage. Thus, brain contusions were predominantly nonsurgical lesions, while epidural hematomas were evacuated immediately in two-thirds of cases.

**Fig. 2:**
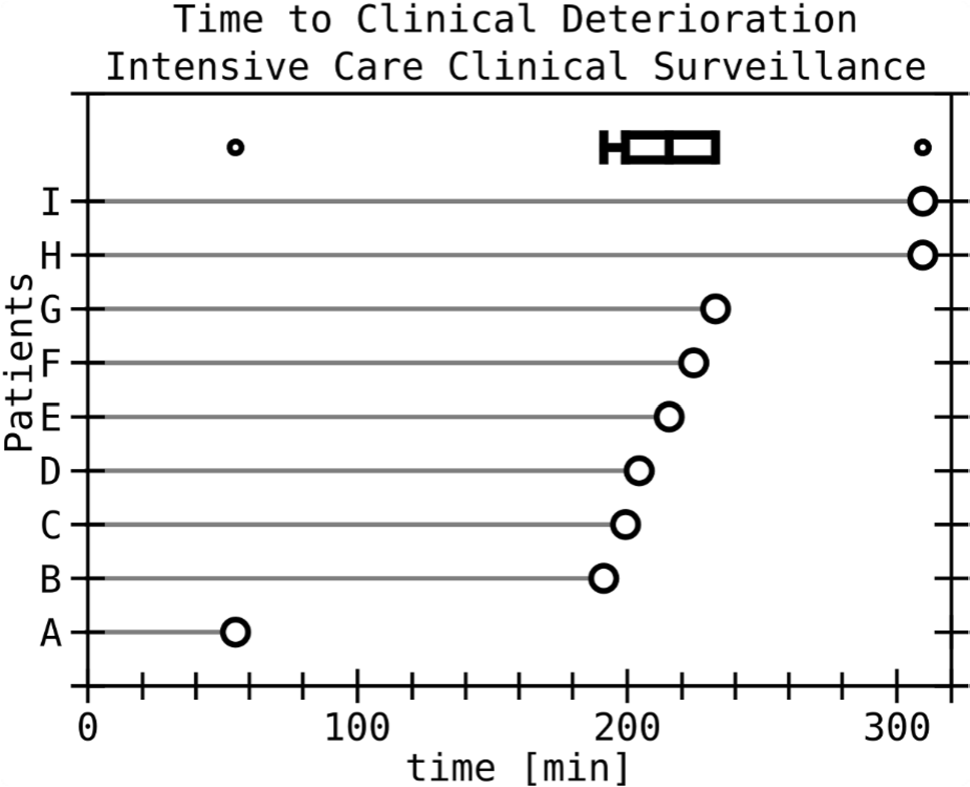
Timing of clinical deterioration under intensive care surveillance before routinely scheduled follow-up CT. Nine patients deteriorated ahead of scheduled CT6h and had emergent CT a median of 216 min after first CT. Patients A to G and I showed sole neurological worsening, while patient H needed intubation due to COVID pneumonia and received an external ventricular drain

One hundred fourteen of 123 patients in the conservative group received CT6h at a median 6 h after first CT, while nine patients underwent earlier CT (mean time to CT 216 min, see Fig. 2) due to neurological deterioration. All nine patients with neurological deterioration underwent immediate surgery, while another eight patients (8/114, 7%) that received the CT6h underwent surgery as well. Of the remaining 106 patients that were transferred to neurosurgical wards or continued to be monitored on the intensive care unit depending on their clinical status, another nine patients (9/104, 8.7%) underwent delayed surgery after two or more follow-up CTs between 25 h and 1 month after admission leaving 97 out of 123 (78.9%) patients initially treated conservatively to definitive conservative treatment.

### Impact of CT6h on surgical decision making

**Fig. 3:**
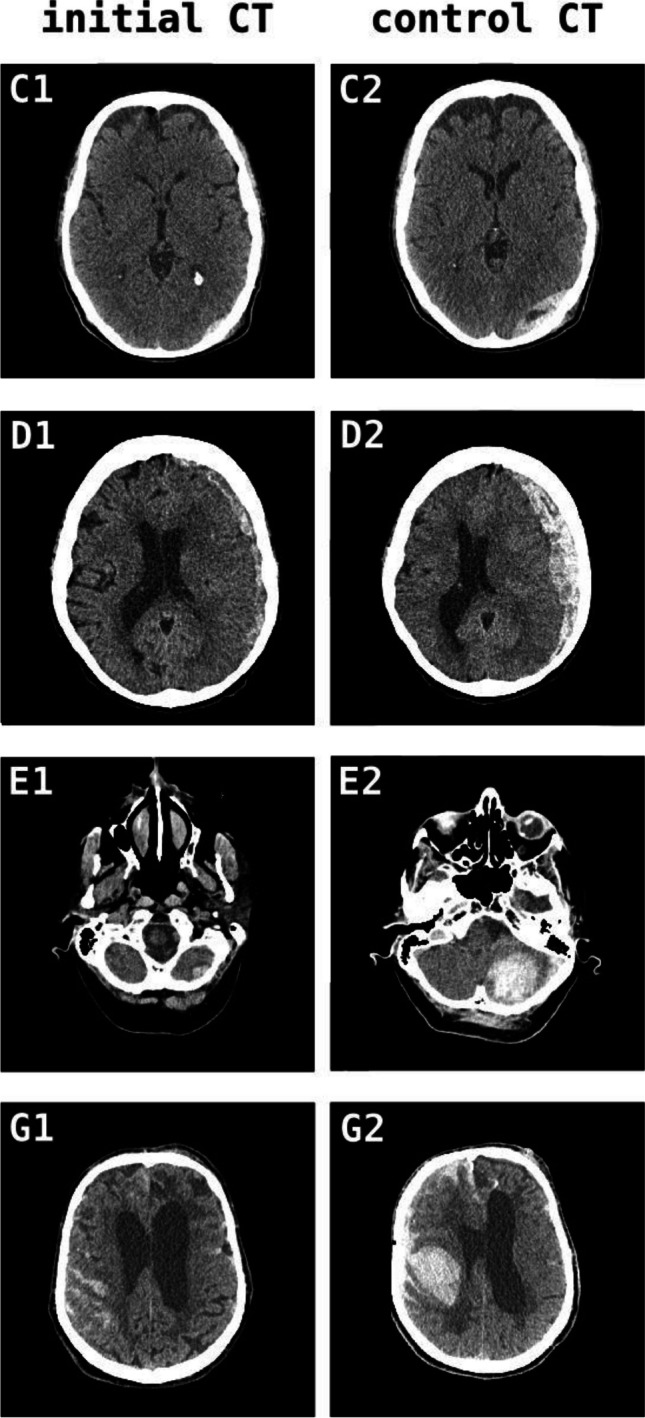
Examples of first and second cranial CT in patients that deteriorated before CT6h and received emergent imaging. The letters at the top left corner of images correlate with the patient identifier in Fig. 2. All patients that showed early deterioration before CT6h received surgical treatment. Left column: CT at admission, right column: emergent follow up CT. CT, cranial computerized tomography. (For illustrative purpose, CT slices with greatest hemorrhage extension are shown. Therefore, slices of initial and control CT might not be at the same level)

Of 123 patients that were initially treated conservatively and monitored clinically, 17 patients were operated after the first follow-up CT (9 had earlier CT due to clinical deterioration, 8 after CT6h). Of note, all patients that deteriorated neurologically (9/17) did so before completion of the 6-h interval and received an earlier CT than CT6h. The timing of clinical deterioration after first CT is displayed in Fig. 2 and shows a clustering between 3 and 4 h after initial CT (mean time to control CT due to clinical deterioration 216 min), while exemplary CT images are displayed in Fig. 3. Eight of nine patients deteriorated in terms of GCS or a new neurological deficit, whereas one patient (patient H in Fig. 3) had to be intubated due to respiratory insufficiency and COVID pneumonia and received an external ventricular drain for intracranial pressure monitoring.

Detailed chart review for the eight patients that were operated on after CT6h revealed that in seven out of eight cases, surgery was indicated after the first CT but scheduled for the following morning usually due to subacute or acute-on-chronic traumatic subdural hematoma. In these cases, CT6h was ordered simply for updating CT imaging before surgery, detected no change, and did not impact the intended treatment. Only in one case (1/114) routine follow-up CT led to a change in treatment protocol since it displayed a vastly different intracranial pathology than initial CT due to poor quality of the initial examination and correction of radiological artifacts.

The nine patients that were operated days to weeks after first and second CT did so due to various reasons ranging from self-dismissal against medical advice (*n* = 1), delayed cerebral edema after 3 and a half days (*n* = 1), delayed onset of headache or neurological deficits after several days (*n* = 4), an initially stable situation under anticoagulation that permitted a conservative treatment attempt with correction of coagulation disorders (*n* = 1), and a failed conservative treatment attempt in elderly with severe comorbidities and subdural chronification (*n* = 2). Of note, out of nine patients that received late surgery, eight had a stable CT6h (four times acute subdural hematoma in combination with contusions or traumatic subarachnoid hemorrhage or frontobasal fracture, five times subacute subdural hematoma).

In summary, only one out of 114 CT6h led to a change in surgical decision making due to poor quality of the initial examination. On the other hand, CT6h did not catch a single imminent neurological deterioration. On the contrary, all patients with neurological deterioration under clinical monitoring worsened before completion of the 6-h interval and were detected clinically. The timing of deterioration clustered between 3 and 4 h after initial CT. Additionally, eight out of nine late deteriorations/indications for surgery had an initially stable CT6h. The predictive value of CT6h for change in surgical management was < 1%. The negative predictive value for later surgical therapy after CT6h was 91.5%, meaning that 8.5% of patients without initial surgical therapy suffered from late clinical deterioration or failure of conservative treatment.

### Impact of CT6h on surgical decision making in mild traumatic brain injuries without major neurological deficit

Since follow-up CT is most controversial in patients presenting with mild TBI (GCS 13 to 15) without any major neurological deficit, they were analyzed separately in this study. Notably, in our clinical routine in an emergency setting, not all patients were screened for subtle neuropsychological deficits. Therefore, only the absence of major neurological deficits can be described by us. One hundred three out of 213 (48.4%) patients presented with an intracranial pathology in initial cranial CT at admission, but with GCS 13 to 15 and without any major neurological deficit. Fifteen out of 103 (14.6%) received immediate surgery due to extension of pathologies alone (including multiple injuries per patient four epidural hematomas, twelve subdural hematomas), and a further six patients (6/103, 5.8%) were operated after CT6h (one EDH, two SDH, one contusion with tSAH, one contusion only, and one tSAH with concomitant depressed calvarial fracture). Again, in half of these patients (3/6), the follow-up CT was carried out earlier than planned due to early clinical deterioration, while in the other three cases, surgery was indicated after the first CT but scheduled for the following morning. In these cases, CT6h was ordered simply for updating CT imaging before surgery, detected no change, and did not impact treatment. However, of the nine patients operated on after days to weeks as described above (see Fig. 1), eight patients presented initially with GCS 13 to 15 without any neurological deficit. Seventy-three out of 103 patients (70.9%) went on to definitive conservative treatment (the remaining 9 patients dropped out due to limitation of therapy by family or patient’s will). This confirms that no imminent deterioration was anticipated by CT6h. Further, it demonstrates that even patients with mild TBI without any major neurological deficit but intracranial pathology have a risk for surgically relevant pathology (21/103, 20.4% after first follow-up CT) and clinical deterioration necessitating monitoring.

### Impact of coagulation disorders on intracranial injury at admission and follow-up

As shown in Table 1, management of coagulation disorders is increasingly relevant in neurosurgery since approximately 50% of patients with TBI and intracranial pathology present with coagulation disorders. Approximately 38% of patients presented to the emergency department with a prior medical history of anticoagulation medication. In our cohort, acetylsalicylic acid (40/213, 18.8%) and direct oral anticoagulation drugs were the most common anticoagulation medication (27/213, 12.7%) to be dealt with, whereas other antiplatelet drugs (9/213, 4.2%) and vitamin K antagonists (8/213, 3.8%) were less often. Additionally, 32% of patients presented with abnormal laboratory testing to various reasons regularly overlapping with the intake of anticoagulation medication.

**Fig. 4:**
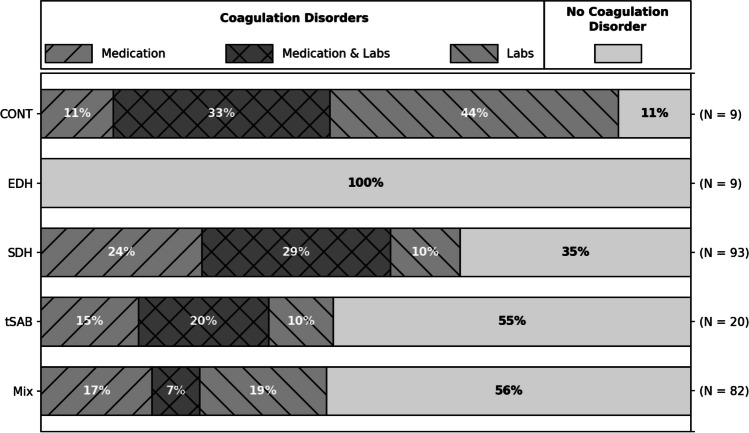
Distribution and type of coagulation disorder across injury patterns. Percentage of coagulation disorder due to medication (anticoagulants/antiplatelet drugs), labs (abnormal laboratory parameters without responsible medication), and both in patients with isolated contusions (CONT), epidural hemorrhages (EDH), subdural hemorrhages (SDH), traumatic subarachnoid hemorrhage (tSAH), or combinations thereof (MIX). This graph illustrates that about half of all patients presented with coagulation disorders. While isolated contusions had a high association with coagulation disorders, epidural hematomas are known to be more associated with the trauma mechanism

**Fig. 5:**
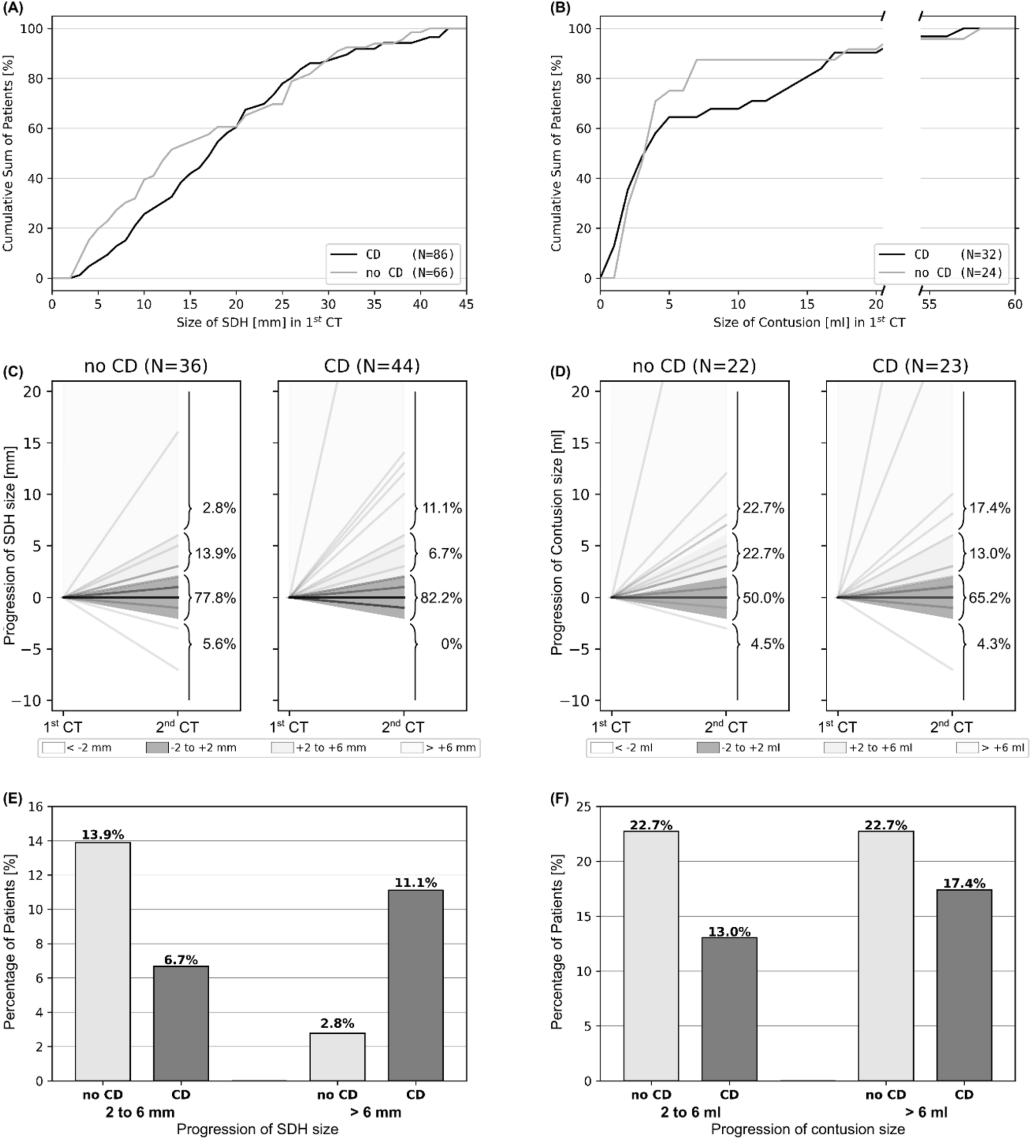
Cumulative size distribution for maximal diameter of SDH (**A**) and volume of contusions (**B**) in any constellation showed no significant differences between patients that presented with (*CD*) or without coagulation disorders (*no CD*) in the first CT scan. Size progression in mm for SDH (**C**) and ml for contusions (**D**) between first and second CT was classified into four groups (< *− 2 mm/ml*: decrease/redistribution, − *2 to* + *2 mm/ml*: stable, + *2 to* + *6 mm/ml*: small increase, >  + *6 mm/ml*: high increase). For patients that showed radiological progression of SDH as part of combined brain injuries under management of coagulopathy (**E**), those with coagulation disorders (CDs) showed more often higher increase of lesion size compared to patients without CDs (diameter increase > 6 mm: 11.1% vs. 2.8%). This effect was not observed for contusions (**F**). Please note that patients who were operated after 1st CT do not show up in the graphs **C**, **D**, **E**, and **F**

The distribution of coagulation disorders across patients with different injury patterns is displayed in Table 2 and Figs. 4 and 5 (anticoagulant medication, anticoagulant medication, and altered coagulation laboratory testing, altered coagulation laboratory testing without anticoagulant medication, and no detected coagulopathy). Of note, none out of 9 patients that presented with isolated epidural hematoma suffered from a coagulation disorder, highlighting this lesion association with a specific injury mechanism, while eight out of nine patients with isolated traumatic brain contusions (without traumatic subarachnoid hemorrhage) showed a impaired coagulation (4/9 taking anticoagulation drugs, 7/9 displaying abnormal laboratory testing). Patients with subdural hematoma (64.5% with coagulation disorder) and traumatic subarachnoid hemorrhage (45% with coagulation disorder) as well as patients with combined brain injuries (43.9% with coagulation disorders) showed ratios of coagulation disorders approximating 50% as was the mean percentage across all injury patterns. Specific analysis of mean volume (contusions) or mean diameter (subdural hematoma) of intracranial pathology revealed that patients with coagulation disorders showed no significant difference in mean lesion size at presentation in isolated subdural hematoma (23 ± 9.9 mm without coagulopathy vs. 20 ± 9.8 mm with coagulopathy on first CT across all therapeutic groups, *p* = 0.16) or in subdural hematoma combined with other traumatic lesions (15.7 ± 10.9 mm without coagulopathy vs. 17.7 ± 9.9 mm with coagulopathy *p* = 0.24) or contusions in combined brain injuries (9.9 ± 15.2 ml with coagulopathy vs. 6.2 ± 11.6 ml without coagulopathy, *p* = 0.32). Of note, there was no difference in mean lesion size in patients with antiplatelet agents versus anticoagulants.

**Table 2:** Characterization of coagulation disorders across injury patterns (left) and according to initial treatment (right). Isolated injuries: *Cont*, contusions; *EDH*, epidural hemorrhage; *SDH*, subdural hemorrhage; *tSAH*, traumatic subarachnoid hemorrhage; *Mix*, any combination thereof. *PC*, platelet count; *Quick*, partial thromboplastin time according to Quick; *INR*, international normalized ratio; *aPTT*, activated partial thromboplastin time. *ASA*, acetylsalicylic acid; *DOAC*, direct oral anticoagulants; *VKA*, vitamin K antagonists

	CONT	EDH	SDH	tSAH	Mix	Surgery after 1st CT	Surveillance and Drop Out
*N*	9/213 [4.2%]	9/213 [4.2%]	93/213 [43.7%]	20/213 [9.4%]	82/213 [38.5%]	78/213 [36.6%]	135/213 [63.4%]
Age	64.0 ± 17.4	36.7 ± 18.7	74.2 ± 13.9	57.4 ± 19.5	61.1 ± 19.9	67.6 ± 18.7	64.3 ± 20.0
Sex (m:f)	3.5: 1	3.5: 1	1.9: 1	1.2: 1	1.7: 1	1.8: 1	1.9: 1
Coagulation disorders	8/9 [88.9%]	0/9 [0.0%]	60/93 [64.5%]	9/20 [45.0%]	36/82 [43.9%]	42/78 [53.8%]	71/135 [52.6%]
Laboratory findings	7/9 [77.8%]	0/9 [0.0%]	37/93 [39.8%]	6/20 [30.0%]	22/82 [26.8%]	25/78 [32.1%]	47/135 [34.8%]
PC < 150/nl	2/9 [22.2%]	0/9 [0.0%]	12/93 [12.9%]	1/20 [5.0%]	9/82 [11.0%]	10/78 [12.8%]	14/135 [10.4%]
Quick < 78% / INR > 1.25	6/9 [66.7%]	0/9 [0.0%]	25/93 [26.9%]	5/20 [25.0%]	17/82 [20.7%]	18/78 [23.1%]	35/135 [25.9%]
aPTT > 38 s	3/9 [33.3%]	0/9 [0.0%]	13/93 [14.0%]	3/20 [15.0%]	11/82 [13.4%]	5/78 [6.4%]	25/135 [18.5%]
Medication	4/9 [44.4%]	0/9 [0.0%]	50/93 [53.8%]	7/20 [35.0%]	20/82 [24.4%]	29/78 [37.2%]	52/135 [38.5%]
ASA	1/9 [11.1%]	0/9 [0.0%]	27/93 [29.0%]	4/20 [20.0%]	11/82 [13.4%]	18/78 [23.1%]	25/135 [18.5%]
Other antiplatet drugs	0/9 [0.0%]	0/9 [0.0%]	8/93 [8.6%]	1/20 [5.0%]	3/82 [3.7%]	5/78 [6.4%]	7/135 [5.2%]
Heparin and derivates	0/9 [0.0%]	0/9 [0.0%]	2/93 [2.2%]	0/20 [0.0%]	2/82 [2.4%]	2/78 [2.6%]	2/135 [1.5%]
DOAC	1/9 [11.1%]	0/9 [0.0%]	18/93 [19.4%]	3/20 [15.0%]	5/82 [6.1%]	7/78 [9.0%]	20/135 [14.8%]
VKA	2/9 [22.2%]	0/9 [0.0%]	5/93 [5.4%]	0/20 [0.0%]	2/82 [2.4%]	3/78 [3.8%]	6/135 [4.4%]

Development of lesion size in all patients with subdural hematoma or contusions undergoing initial control imaging is displayed graphically in Fig. 5, while the ratio of patients with relevant increase in lesion size is additionally shown in Table 3. In summary, 16.7% (6/36) of patients with subdural hematoma without coagulopathy showed radiological progression of more than 2 mm increase in hematoma diameter, while 17.8% (8/45) of patients with subdural hematoma with coagulopathy (under institutional standard-of-care clinical management of coagulation disorders) presented this same radiological progression. Of note, while hematomas that showed radiological progression between 2 and 6 mm were predominantly in patients without coagulopathy (13.9% of patients without coagulopathy vs. 6.7% of patients with coagulopathy), an increase of more than 6 mm was mostly in patients with coagulopathy (11.1% of patients with coagulopathy vs. 2.8% of patients without coagulopathy) as displayed in Fig. 5. This effect does not pertain to contusions.

**Table 3:** Development of lesion size. Left: lesion size at first CT in mm for patients with SDH in any combination of injuries or traumatic brain contusions in any combination of injuries as means with standard deviation in mm for SDH and ml for contusions (*no CD*, no coagulation disorder; *CD*, coagulation disorder). Right: development of lesions from first to second CT with ratio of lesions decreasing in size > 2 mm, remaining stable, increase in size of 2–6 mm or more than 6 mm (ml for contusions)

	1st CT		2nd CT	Dynamic 1st–2nd CT			
	SDH size [mm]	*N*	*N*	Decrease (< − 2 mm)	Stable (+ / − 2 mm)	Increase	
						(2–6 mm)	(> 6 mm)
SDH no CD	15.7 ± 10.9	66	36	2/36 [5.6%]	28/36 [77.8%]	5/36 [13.9%]	1/36 [2.8%]
SDH CD	17.7 ± 9.9	86	45	0/45 [0.0%]	37/45 [82.2%]	3/45 [6.7%]	5/45 [11.1%]
	CONT size [ml]	*N*	*N*	Decrease (< − 2 ml)	Stable (+ / − 2 ml)	Increase	
						(2–6 ml)	(> 6 ml)
CONT no CD	6.2 ± 11.6	24	22	1/22 [4.5%]	11/22 [50.0%]	5/22 [22.7%]	5/22 [22.7%]
CONT CD	9.9 ± 15.2	32	23	1/23 [4.3%]	15/23 [65.2%]	3/23 [13.0%]	4/23 [17.4%]

## Discussion

### Impact of early follow-up CT on surgical decision making

The following observations are made in this retrospective study: Firstly, CT6h did not anticipate imminent neurological deterioration. Contrarily, all patients that showed early neurological deterioration (9/123, 7.3%) did so before the completion of 6 h after admission with a marked clustering between 3 and 4 h after admission (6/9). This highlights the importance of clinical surveillance to detect early neurological deterioration which cannot be substituted with routine imaging. Secondly, in patients that received CT6h, only one case (1/114) changed surgical decision making by correcting a false evaluation of initial CT due to artifacts. Thirdly, out of all patients that went on to conservative management after CT6h (*n* = 106), nine patients (8.5%) demonstrated a late clinical deterioration or failure of conservative treatment after days, eight out of which had a stable CT6h. These findings suggest that CT6h in patients that can be monitored clinically does not serve to anticipate imminent neurological deterioration and does not relevantly impact surgical decision making. Additionally, an initially CT6h may suggest stability of pathology, although 8.5% of patients need delayed surgical intervention due to delayed deterioration. Thus, in all scenarios in patients that can be monitored clinically, surgical decision making depends on clinical status instead of imaging (7.3% clinical deterioration versus 16.7 to 45.5% of radiological progression). However, follow-up imaging may nevertheless impact conservative management such as length of stay on an intensive care unit for close continuous neurological monitoring or timing of thrombosis prophylaxis [14].

These findings support the results of Sifri et al. who reported on repeat CT within 24 h after minimal TBI with intracranial pathology a negative predictive value of a normal neurological exam for surgical intervention of 100%, while out of 31 patients with an abnormal exam, 45% worsened at repeat scan [11]. Consequently, the authors questioned the diagnostic value of repeat CT without neurological deterioration. Brown et al. both retrospectively and prospectively studied the impact of follow-up CT after an average of 38 h in patients with blunt head trauma and abnormal initial head CT on the frequency of surgical intervention [9, 10]. They also stated that while roughly 90% of repeat CT scans are carried out in patients without any neurological worsening, only 1% lead to a new medical or surgical intervention. On the other hand, 38% of repeat scans due to neurological deterioration led to a new medical or surgical intervention. Thus, the authors concluded that follow-up CT scans impact treatment only in patients with GCS of eight or less to substitute clinical monitoring or with neurological deterioration. Similar findings were presented among others by Ding et al., Haider et al., and Connon et al. However, in all these studies, routine follow-up imaging was carried out within 24 to 48 h [15–17]. This naturally creates a bias towards only stable patients receiving routine follow-up CTs, while early clinical deterioration is routinely missed leading to a potentially premature conclusion that follow-up CTs do not influence treatment protocols. However, in our investigation of a routine follow-up CT protocol with decidedly early repeat scans after 6 h, surgical decision making was still almost exclusively based in neurological status, while early follow-up CT still missed all early neurological deteriorations. Nevertheless, in the case of unconscious patients that cannot be monitored clinically, management driven by repeat CT scans was shown to have an outcome similar to ICP-driven management [18].

### Impact of early follow-up CT on surgical decision making in mild traumatic brain injuries without neurological deficit

Ding et al. carried out a prospective study comparing change in treatment protocols after routine follow-up CT and repeat CT scan due to neurological deterioration in 172 patients that were not treated surgically initially [16]. They reported that whereas 18.4% of routine repeat scans changed treatment in moderate and severe traumatic brain injury, 0% changed treatment in mild traumatic brain injury. Similarly, AbdelFattah et al. demonstrated that a highly selective use of repeat CTs in TBI patients with GCS 13 to 15 and intracranial hemorrhage does not lead to a worse outcome but shortens hospital length of stay, while the reduction of repeat CTs did not lead to an increase in change of therapy [12]. Because the largest amount of patients with TBI presents with GCS 13 to 15, classifying as mild TBI, the value of early follow-up CT in this group is of particular significance in day-to-day clinical practice and was analyzed separately in this study. While no routine follow-up CT after 6 h led to a change in surgical decision making, it is noteworthy that even in this allegedly mildly affected group of patients, three early (3/103 after exclusion of surgical treatment after first CT, 2.9%) and eight late clinical deteriorations (8/97 after exclusion of surgical treatment after first and second CT, 8.2%) occurred. This highlights the potential for secondary deterioration and the need for close observation and monitoring even in mild TBI as well as proposed by Anandalwar et al. [8].

### Impact of coagulation disorders on intracranial injury at admission and follow-up

TBI is a known cause of coagulopathy. Vice versa, coagulopathy increases the risk of intracranial hemorrhage and hemorrhagic progression in patients with traumatic brain injury [19]. With the increasing age of patients with TBI, preinjury intake of antiplatelet agents and anticoagulants is a common problem [20]. While the impact of preinjury antiplatelet agents in TBI may increase unfavorable outcome but is poorly understood, oral anticoagulants are well known to increase mortality with vitamin K antagonists displaying a higher risk than direct oral anticoagulants [21–23]. Coagulopathy appears to be particularly associated with the incidence and progression of intracerebral hemorrhage in contrast to extraaxial traumatic lesions [24]. Notably, in this study, coagulopathy led to a larger ratio of patients with an increase in SDH size > 6 mm in repeat CT (11.1%) compared to patients without coagulopathy (2.8%). However, there was no difference in the hemorrhagic progression of contusions depending on the presence of coagulopathy. Radiological progression generally was most prevalent among traumatic brain contusions (17.8%) as has been described by Alahmadi et al. in up to 45% of cases [25]. Conversely, neither patients with subdural hematoma nor contusions displayed different hematoma diameters at presentation depending on coagulation status.

### Limitations

The main limitations of this study are rooted in its retrospective nature and the heterogenous character of TBI. Further implicit bias is given, because only patients that were admitted to our neurosurgical department were included in this analysis. In our hospital, patients with mild TBI (GCS 13–15) without major neurological deficits but minor radiological findings and concomitant trauma were admitted to our trauma surgery department. If the concomitant trauma was a spine trauma, these patients were admitted to neurosurgery. The patients admitted to trauma surgery underwent the same standardized guideline-compliant conservative TBI management as described above. In case of unexpected deterioration, these patients were treated by neurosurgery. Anyway, if the patients admitted to trauma surgery would be included in this study, the percentage of relevant findings in follow-up CTs would likely be further decreased.

In over 99.5% of the cases (excluding the 12 dropouts), TBI management was in accordance with our guidelines. The authors point out that especially in cases of psychiatric comorbidities and/or intoxication ICP monitoring was not always forced, but spontaneous improvement was awaited. The sample size reflects all neurosurgically admitted patients fulfilling the above-mentioned criteria in a level I trauma center in Berlin, Germany, over a period of 2.5 years. Further multicenter studies might be needed to confirm our results.

## Conclusion

In all scenarios in patients that can be monitored clinically, surgical decision making depends on clinical status instead of imaging. However, follow-up imaging may nevertheless impact conservative management such as length of stay on an intensive care unit for close continuous neurological monitoring or timing of thrombosis prophylaxis [14]. Furthermore, no difference in lesion size at presentation according to the presence of coagulopathy was detected in subdural hematomas or traumatic brain contusions. SDH with coagulopathy showed a higher percentage of drastic increase (> 6 mm: 11.1% with vs. 2.8% without, 2–6 mm: 6.7% with vs. 13.9% without coagulopathy).
